# The Theories of Gerbrandus Jelgersma (1859–1942) on the Function of the Cerebellum

**DOI:** 10.1007/s12311-021-01273-4

**Published:** 2021-08-12

**Authors:** Jan Voogd

**Affiliations:** Department Neuroscience, Erasmus MC, Rotterdam, The Netherlands

**Keywords:** Neurons: amoeboid movements, Purkinje cell: double innervations, Cerebellum: anatomy, Golgi Cox method, Marr and Albus cerebellar theory, Leiden University

## Abstract

Gerbrandus Jelgersma published extensively on the (pathological) anatomy of the cerebellum between 1886 and 1934. Based on his observations on the double innervation of the Purkinje cells, he formulated a hypothesis on the function of the cerebellum. Both afferent systems of the cerebellum, the mossy fiber-parallel fiber system and the climbing fibers terminate on the Purkinje cell dendrites. According to Jelgersma, the mossy fiber-parallel fiber system is derived from the pontine nuclei and the inferior olive, and would transmit the movement images derived from the cerebral cortex. Spinocerebellar climbing fibers would transmit information about the execution of the movement. When the Purkinje cell compares these inputs and notices a difference between instruction and execution, it sends a correction through the descending limb of the superior cerebellar peduncle to the anterior horn cells. Jelgersma postulates that this cerebro-cerebellar coordination system shares plasticity with other nervous connections because nerve cell dendritic protrusions possess what he called amoeboid mobility: dendritic protrusions can be extended or retracted and are so able to create new connections or to abolish them. Jelgersma’s theories are discussed against the background of more recent theories of cerebellar function that, similarly, are based on the double innervation of the Purkinje cells. The amoeboid hypothesis is traced to its roots in the late nineteenth century.

In this paper, I will trace the ideas on the anatomy of the cerebellum of Gerbrandus Jelgersma (1859–1942, Fig. 1) that culminated in his hypothesis on the function of the cerebellum and the plasticity of its connections. One of the main challenges of a hypothesis on the cerebellum’s function should be that it explains the double innervation of the Purkinje cells by the mossy fiber-parallel fiber system and the climbing fibers, as illustrated in Fig. 2. Mossy fibers branch and terminate on granule cells in the granular layer of the cerebellar cortex. Granule cells give rise to an axon that ascends to the molecular layer where it bifurcates into the parallel fibers. These fibers terminate on the spiny branches of the Purkinje cell dendrites. Climbing fibers climb and terminate on the smooth proximal branches of these dendrites. Jelgersma’s hypothesis is one of the first attempts to explain the function of this double innervation.

**Fig. 1:**
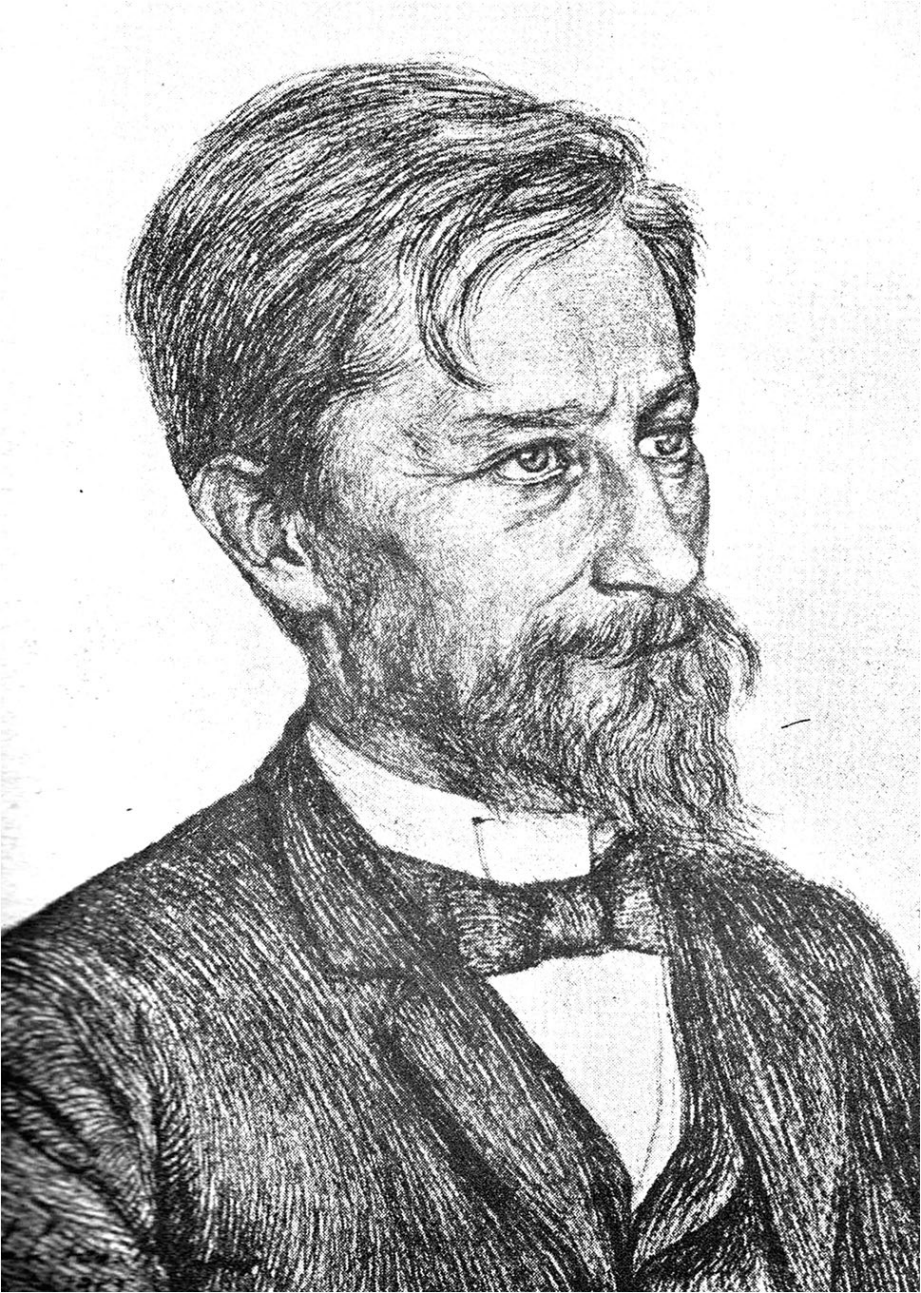
Jelgersma as a young professor. Drawing by van Manen. Carp [1] Reproduced from

**Fig. 2:**
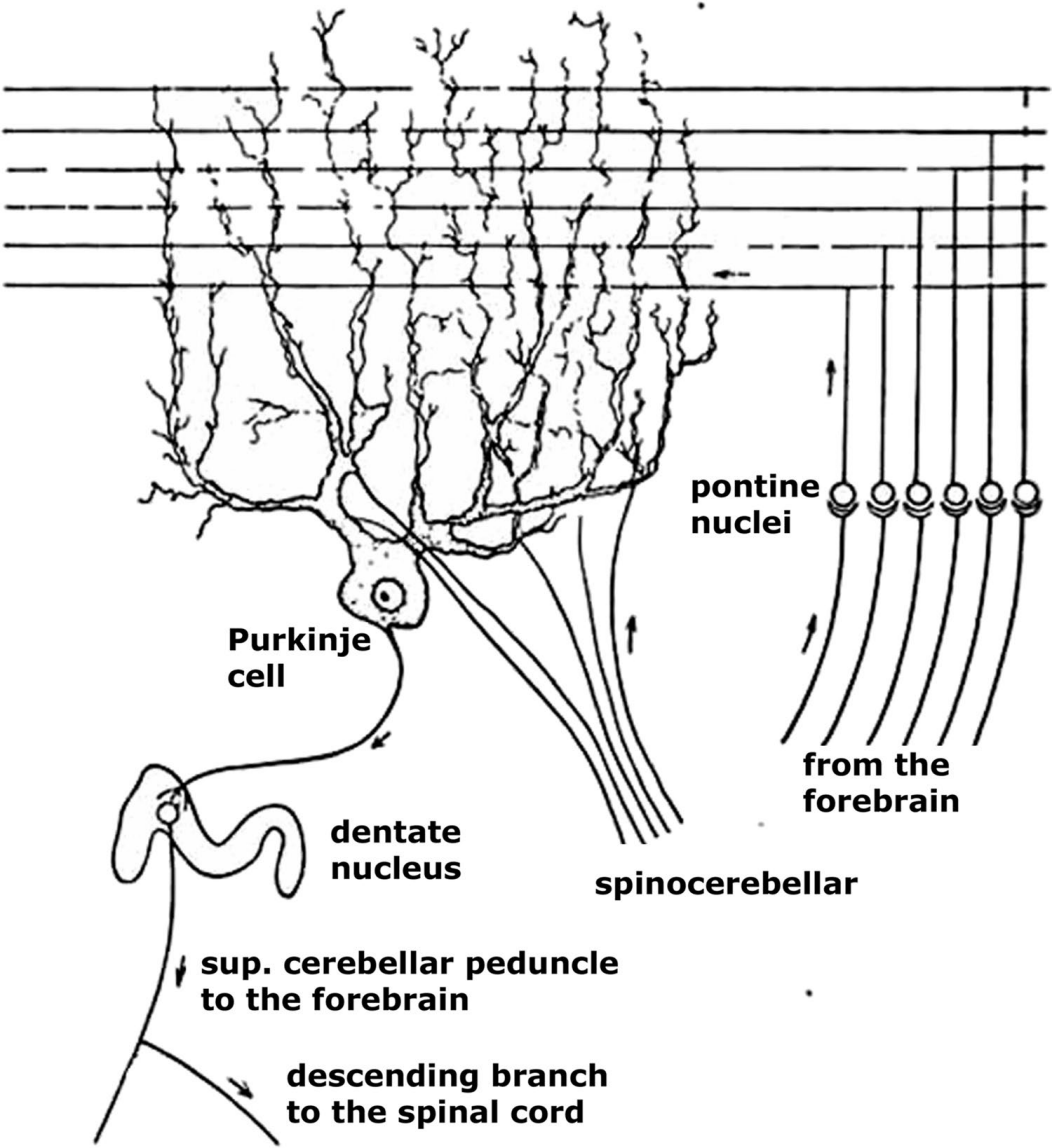
Jelgersma’s [2] diagram of the double innervation of the Purkinje cells by the mossy fiber-parallel fiber system from the pontine nuclei and the climbing fibers as terminations of the spinocerebellar tract. Lower left the superior crebellar peduncle, issued by the dentate nucleus, is shown, with its descending and ascending branches

Between 1886 and 1934, Jelgersma published three books and numerous papers on the (pathological) anatomy and function of the cerebellum. Most of these publications are in Dutch, some were published as translations in German Journals. A biography of Jelgersma was written by his student and later successor Eugène Carp [1, 2]. His collection of slides of normal and pathological human brains was reviewed by Marani et al. [3].

Jelgersma attended high school in Alkmaar, in North Holland and studied medicine in Amsterdam. In 1877, even before he had obtained his medical degree, he was appointed as prosector in the asylum of Meerenburg, where he was responsible for the autopsies and where he acquired the necessary skills for studying nervous tissue under the microscope [4]. As a staff physician he remained employed by this asylum. He left Meerenburg when he was appointed as a private lecturer in criminal anthropology at the University of Amsterdam and became director of the Arnhem asylum. In 1896 he received an honorary doctorate of the University of Utrecht, together with W.H. Cox. Both had attended highschool instead of the gymnasium that was a requirement for a doctorate at the Dutch universities. Cox was responsible for the introduction of Cajal’s neurohistology in the Netherlands [5, 6] and he developed the well-known Golgi-Cox modification of the Golgi technique [7]. In 1899 Jelgersma became the first professor of psychiatry at the Leiden University. A hospital for his patients, where he had all facilities for neuropathological research, was built for him on the grounds of the asylum Endegeest (Fig. 3). It is situated at a few hundred meters from the old farmhouse on the same grounds where this paper was written. Originally, Jelgersma was faithful to the biologically oriented neuropsychiatry of the late nineteenth century. In later years he got interested in Freud’s ideas as shown by his lecture as the Rector magnificus of Leiden University in 1914, entitled “The unconscious mind”. He used psychoanalysis as part of his psychopathology, but never applied it in his therapy.

**Fig. 3:**
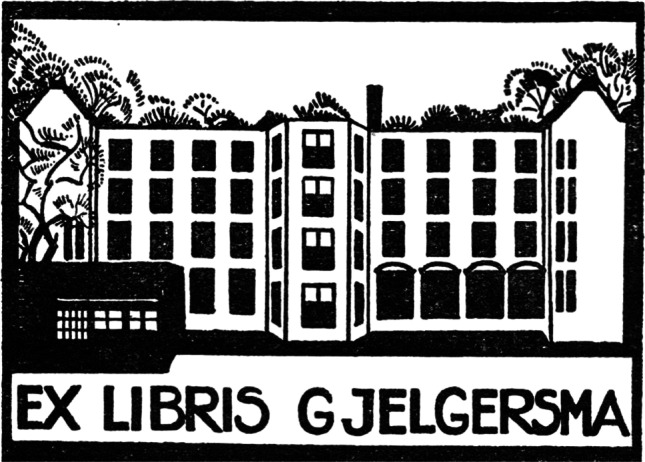
Ex libris from Jelgersma, showing his clinic for patients with a neurosis. Reproduction from Carp [1]

## The intellectual system

**Fig. 4:**
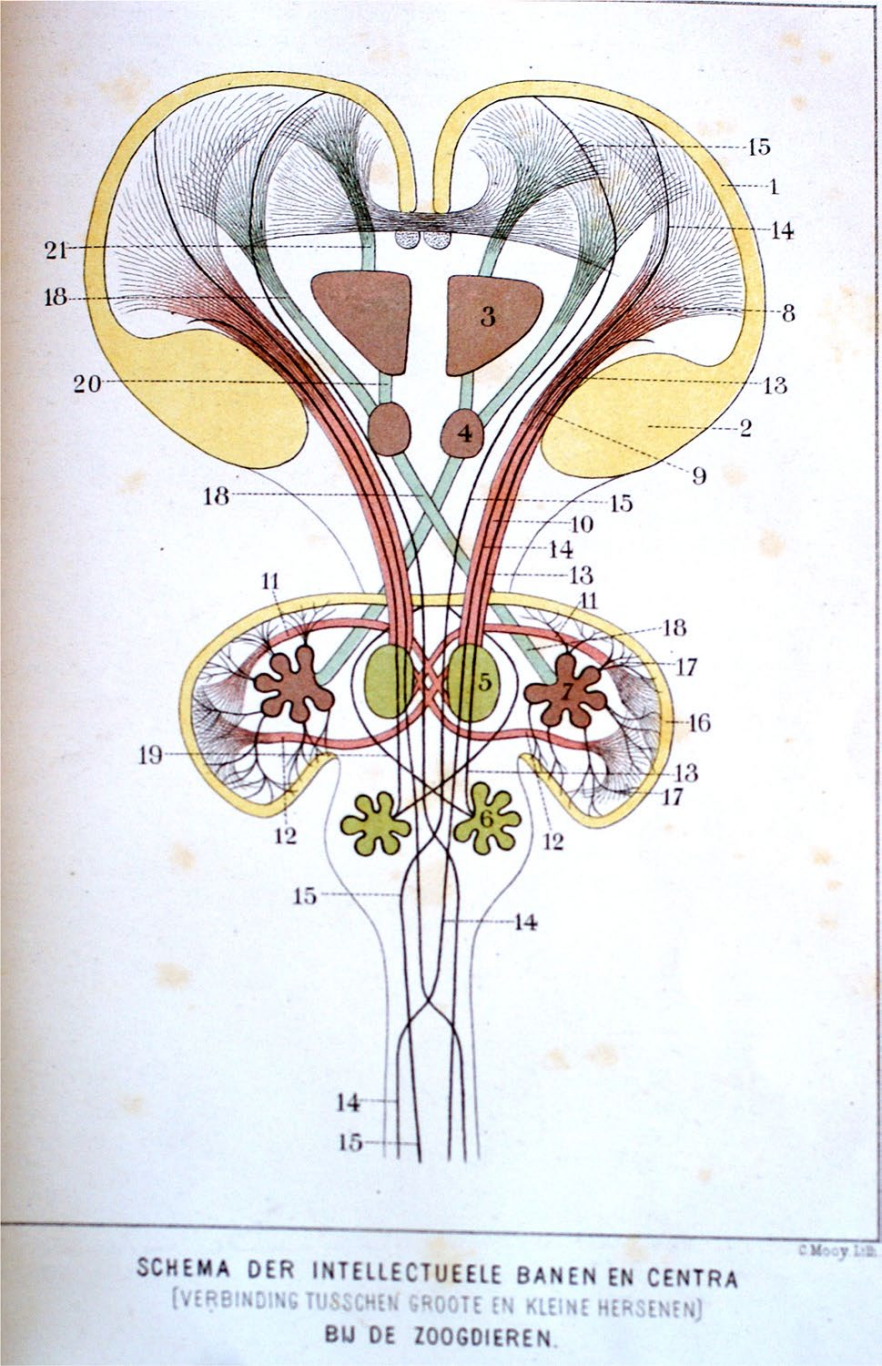
Diagram of the intellectual system of connections; reproduction with permission from Nederlands Tijdschrift voor Geneeslunde, Jelgersma [10]. For legends see text

Jelgersma summarized his early ideas on the anatomy of, what he called “the intellectual system”, the connections between the forebrain (the “intellectuarium”) and the brainstem with the cerebellum [8–13] in a figure (Fig. 4). The system connecting the cerebral hemispheres with the cerebellum is indicated in red. The cerebral cortex (1 in Fig. 4) and the basal ganglia (2) give rise to the internal capsule and the cerebral peduncle (8, 9, 10) that terminates in the ipsilateral pontine nuclei (5), arciform nuclei and inferior olive (6). Pontine and arciform nuclei project via the brachium pontis (11, 12) to the contralateral cerebellar hemisphere. The inferior olive gives rise to arciform fibers that reach the cerebellar vermis via the contralateral restiform body (19). The cerebellar cortex of the hemisphere is connected with the dentate nucleus (7, 17). The superior cerebellar peduncle (18, blue in Fig. 2) takes its origin in the dentate nucleus (7), decussates and terminates mainly in the red nucleus (4). According to Jelgersma, the red nucleus is connected with the cerebral cortex directly as well as via the thalamus (3). Jelgersma mentions the atrophy of the ipsilateral pontine, arciform nuclei and the inferior olive in his cases of crossed cerebro-cerebellar atrophy as evidence for the dependance of these structures on the cerebral hemisperes.

The connection between the basal ganglia and the ipsilateral inferior olive, described as the central tegmental tract by Bechterew [14], is not mentioned by Jelgersma, but included in the cerebral peduncle in his diagram (13). Jelgersma found support for the projection of the basal ganglia, through the inferior olive to the vermis, in the constellation in birds, where he found the entire forebrain to consist of the striatum, while the cerebellum is only composed of a vermis with small auricles possibly representing the incipient hemispheres. The idea of the avian forebrain as the equivalent of the mammalian striatum is based on the composition of both structures of random clusters of nerve cells. As we now know, the equivalent of the mammalian layered cortex in birds consists of clusters of neurons. Jelgersma’s idea on the interruption of the cerebello-cerebral pathway in the red nucleus probably stems from the work of Stilling [15], Flechsig [16], and Bechterew [16]. Forel’s [17] description of the termination of the superior cerebellar peduncle in the thalamus, apparently, was unknown to him. The cerebello-spinal pathway that passes through the brachium pontis, described by Flechsig [18], and mentioned by Jelgersma in his text, is not included in the diagram. It was later confirmed with Marchi’s method for tracing nerve fibers with osmium staining of degenerated myelin [19]. His rather vague conclusion in his 1890 paper “On the function of the cerebellum” reads: “For the moment we consider the cerebral hemispheres and the cerebellum as two coordinated structures”.

## The cerebro-cerebellar coordination system

**Fig. 5:**
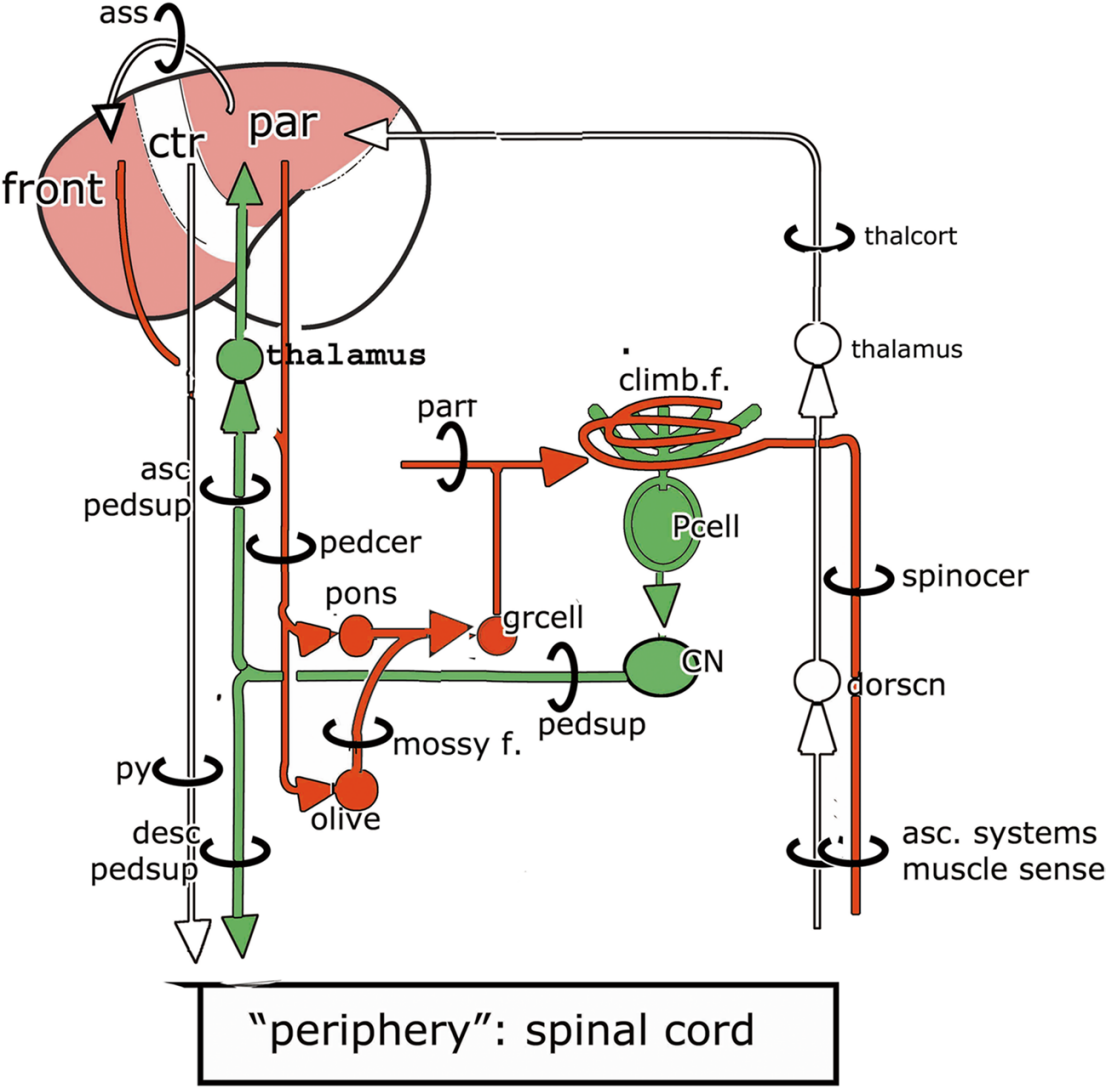
Diagram of Jelgersma’s cerebro-cerebellar coordination system. Abbreviations: ascpdsup, ascending branch superior cerebellar peduncle; ass, cortical association system; climb.f., climbing fiber; CN, cerebellar nuclei; descpedsup, descending branch of the superior cerebellar peduncle; dorscn, dorsal column nuclei; front, frontal lobe; grcell, granule cell; Par, parietal lobe; parr, parallel fiber; Pcell Purkinje cell; pedcer, cerebral peduncle; pedsup, superior cerebellar peduncle; py, pyramidal tract; spinocer, spinocerebellar tract; thalcort, thalamocortical connection

In his publications from the early twentieth century [20–24], Jelgersma made changes in his diagram of the connections of the intellectual system, which was now called the “cerebro-cerebellar coordination system” (Fig. 5). Coordination, as Jelgersma uses the term, stands for the correction of movement images. The cerebellar hemispheres are concerned with “higher coordination”, the correction of complex, learned, voluntary movements, on the basis of proprioceptive and vestibular input. Lower coordination of simple movements is handled by the spinal reflex system [20, 21]. Voluntary movements are learned and stored in the forebrain as movement images. Once learned, the control of voluntary movement is transferred to the cerebellum and thus becomes automatic and subconscious.

The parietal lobe receives both muscle sense from the periphery, and input from the cerebellum. Cortical association systems project movement images from the parietal lobe to the frontal lobe. Here crude motor images are located in the precentral gyrus that gives rise to the pyramidal tract, a tract that conveys these images to the anterior horn cells [22–24]. More complex movement images, which are located more rostrally in the frontal lobe, are connected through Arnold’s fronto-pontine tract in the medial cerebral peduncle to the pontine nuclei. Türck’s corticopontine tract, located in the lateral part of the peduncle, takes its origin from the temporal lobe, which would contain movement images from the vestibular system [21]. According to Reitsma [25], one of Jelgersma’s students, the parietal, rather than the temporal lobe would be the origin of Türck’s tract. The pontine nuclei, therefore, would receive information from all cortical regions involved in movement.

The cerebellar hemisphere projects through the dentate nucleus as the superior cerebellar peduncle to the contralateral ventral thalamus. In this nucleus, this projection is united with the sensory input through the dorsal column nuclei and the medial lemniscus. The ventral thalamic nucleus projects to the parietal lobe. Later,Jelgersma added the frontal and temporal lobes to its targets.In his 1934 paper, the frontal lobe is no longer mentioned as a target. The red nucleus no longer is considered as a link in the cerebello-cerebral pathway [23, 24]. He emphasized that the (magnocellular) red nucleus and its efferent rubrospinal tract in primates becomes reduced with the increase in size of the cerebellar and cerebral hemispheres. Jelgersma does not mention the increase in size of the parvocellular red nucleus or its position as the origin of the central tegmental tract [26, 27]. The crossed descending branch of the superior cerebellar peduncle now takes the place of Flechsig’s direct cerebello-spinal system [28]. Jelgersma supposed that it terminated on anterior horn cells but was not sure of this.

An important addition to the coordination system was his identification of mossy and climbing fibers as the terminals of different afferent cerebellar systems. In a study of kittens with inborn atrophy of the cerebellum [29], which is caused, as we know now, by an intrauterine viral infection [30, 31], Jelgersma noticed the absence of the granular layer of the cortex in the cerebellar hemispheres, whereas the Purkinje cells were relatively spared. There was atrophy of the pontine nuclei and the inferior olive as well. From these observations, he concluded that these centers, which relay complex movement images to the cerebellum, give rise to the mossy fibers that would have terminated in the now defunct, granular layer. The pontine nuclei project to the contralateral hemisphere, while mossy fibers from the olive would terminate in the cerebellar vermis. If the afferent cortico-pontine system terminates as mossy fibers in the granular layer, it is likely that the afferent systems from the periphery terminate as climbing fibers “because the cerebellum only receives two kinds of afferent systems” [23, 23]. There is no other supporting evidence for the termination of the spinocerebellar, trigemino-cerebellar, cuneocerebellar and reticulocrebellar tracts as climbing fibers. Through these climbing fiber systems the cerebellum receives muscle sense (proprioscepsis), as its major sensory input [24, 24]. Vestibulocerebellar climbing fibers would terminate in the vermis. Jelgersma’s conclusion differs from Cajal’s [28] who supposed that ponto- and vestibulocerebellar afferents terminate as climbing fibers and spinocerebellar axons as mossy fibers. Of course direct evidence for the origin of the mossy and climbing fibers was not available at the time.

## Jelgersma’s theory on the function of the cerebellum [32]

Already in his 1920 monograph, Jelgersma suggested that the cerebellum corrects mistakes in movements through contacts between afferent sensory information about the actual movements and the cerebral movement images. In 1932 and 1934, he formulated his theory more precisely. The Purkinje cell is the central element in his theory. An equivalent image of peripheral movements is established in the Purkinje cells through the ascending climbing fiber systems, and an equivalent representation of the cerebral movement image through the corticopontine mossy fiber-parallel fiber system (Fig. 1). When the two images are similar, the movement is made as it was programmed by the cerebral cortex. If there is a disparity between the images a correcting stimulus is sent by the Purkinje cell to the periphery by way of the descending limb of the superior cerebellar peduncle without the forebrain being involved. This subconscious phenomenon would guarantee the required speed for correction of a movement.

## The amoeboid theory of neuronal mobility

In his papers from 1928 “Schakelingen” (Dutch for connections), 1932 and 1934, Jelgersma tried to explain how a single stimulus may result in different reactions, depending on the circumstances, how the nervous system is able to learn new complex movement images and how it is able to correct them. His hypothesis supposes that nerve cells possess a kind of amoeboid motility, with pseudopodia that can be extended or retracted. These protrusions are able to make new connections with axons of other neurons. Different “connectomes” of ganglion cells with other ganglion cells make learning of new movements as well as correction thereof possible. Learning does not depend on new neurons, but on new connections [33]. In this, design functions of neural networks may create an immense complexity. In the cerebro-cerebellar coordination system, these new connections would be established in the afferent, cortico-ponto-cerebellar, and efferent cerebello-cortical systems. Jelgersma does not use Cajal’s [34] term “épines collatérales” (spines) for the protrusions, and fails to mention Sherrington’s [35] term “synapse” for the connections, although he cites both authors in his publications. Did Jelgersma pay sufficient tribute to those who generated the original ideas on amoeboid movements of neurones? He never referred to the theories of the late nineteenth century that suggested that dendritic spines were capable of limited movements of extension and contraction, which would alter interneuronal connections [36, 37].

## Original ideas?

Jelgersma’s theory on the function of the cerebellum is certainly original. It would last till 1969 for David Marr [38] to propose a similar theory about how the cerebellar cortex learns motor skills that is based on more realistic anatomical and phyiological data. Marr’s publication was followed by a similar, more formal, theory by Albus [39]. Jelgersma’s ideas on the origin of mossy- and climbing fibers have proven to be wrong. Climbing fibers originate from the inferior olive, not from the spinal cord. Other afferent cerebellar systems terminate as mossy fibers [27, 40]. His description of the crossed descending branch of the superior cerebellar peduncle as the link in the connection of the cerebellum with the anterior horn cells has not been substantiated [41]. Cerebellar connections with the spinal cord consist of the vestibulo- and reticulospinal tracts [42]. Jelgersma’ papers are exclusively concerned with the cerebellar hemispheres. The vermis, its connections, and possible functions are scarcely mentioned.

In Marr’s theory, each neuron of the inferior olive and the Purkinje cell that receives its climbing fiber from that olivary neuron would respond to an instruction for an elementary movement. Not so much different from Jelgersma’s concept of the equivalent of the movement image derived from the motor cortex, but in this case, carried by the climbing, not by the mossy fibers. This Purkinje cell is exposed, via the mossy fiber–parallel fiber pathway, to information about the context in which the elementary movement is performed. This context would stand for Jelgersma’s equivalent image of peripheral movements. After repeated rehearsal of this action, the Purkinje cell can correct an eventual error in the context, because the parallel fiber to Purkinje cell synapses are facilitated (Marr) or weakened (Albus) by the conjunction of parallel fiber and climbing fiber activity.

Marr’s ideas were applied by Ito [43] in his flocculus hypothesis for long-term adaptation of the vestibulo-ocular reflex (VOR). Ito’s hypothesis has led to intensive research on the adaptation of the VOR and the related question of the contribution of the cerebellum to the learning of motor skills. Ito’s theory has been supported, but also received serious critisism. It was reviewed in a recent paper [44]. A similar example of “higher coordination” by the cerebellum is found in the adaptation of saccades by lobule VII of the cerebellar vermis [45]. Eyeblink conditioning by the cerebellum was also found to be based on the double innervation of the Purkinje cells, with the conditional stimulus representing the context, transmitted by the mossy fiber-parallel fiber input and the unconditional stimulus, representing the elementary movement, transmitted by the climbing fibers [46]. Indeed, the lattice structure of the cerebellar cortex allows the pairing of a conditinal stimulus with an immense variety of unconditional stimuli.

Both Eling [4], in his short biography of Jelgersma and I in several historical reviews of cerebellar research [47],Voogd and Koehler [48], emphasized Jelgersma’s theory of amoeboid mobility of nerve cells, a theory leading to a self-generating neural network that subserves neuronal plasticity. However, reading more about the history of neuronal spines [49], I found that Jelgersma never mentioned that this theory was initiated by a group of Belgian and French histologists in the late nineteenth century. Their work was extensivey reviewed by Binet [50] and Black [51].

The first mention of the term amoeboid movements of neurons was in a paper of Rabl-Rückhart [37] “Sind die Ganglienzelle amöboid?” (Are ganglien cells amoeboid?). These amoeboid movements would reside in Golgi’s network of protoplasmic (dendritic) processes of neurons. “The psychic world resides in this neurospongium”. “Protoplasmic processes may vary in their contacts. Amoeboid movements of these branches would offer the mechanical explanation of psychic phenomena. With the interruption of protoplasmic branches, memories would be lost”. According to Black [51], the amoeboid theory was the consequence of Cajal’s evidence for the neuron theory that focused the interest on the place of linkage between neurons. The gap between the neurons at the place of the junction was regarded as a resistance in the conduction of the impulse that would vary with the size of the gap. Duval’s [36] amoeboid theory offered an explanation of this phenomenon. He proposed that the ramifications of nerve cells would be able to extend or retract due to veritable amoeboid properties of their protoplasm. Under different circumstances the degree of contiguity between neurons may differ. The amoeboism of nervous pseudopodia that would approximate each other, and thus facilitate passage of the impulse, would explain processes as imagination, memory, association of ideas and the effects of stimulants like coffee. Sleep would occur when sensory neurons would temporarily retract their ramifications, abolishing the reception of peripheral stimulation. Duval refers to an ealier suggestion of Lépine [49, 52] for this explanation of the state of sleep.

Experimental support for the theory was obtained by scientists from the Institut Solvay in Brussels, who found distinct changes in Golgi-stained dendrites under different experimental conditions. Jean Demoor [53] showed that in dogs subjected to morphine or chloroform the dendrites of cortical pyramidal cells showed a beaded (moniliform, “état perlé”) appearance, disrupting interneuronal conduction, an observation very similar to the changes in pseudopodia of amoebas when subjected to anaesthetics.

Using the same method, Micheline Stefanowska [6] showed that electrical stimulation caused dendritic spines (her “appendices piriformes”) of pyramidal neurons in mice and guinea pigs to expand or retract, thus interfering with neuronal conduction (Fig. 54).

**Fig. 6:**
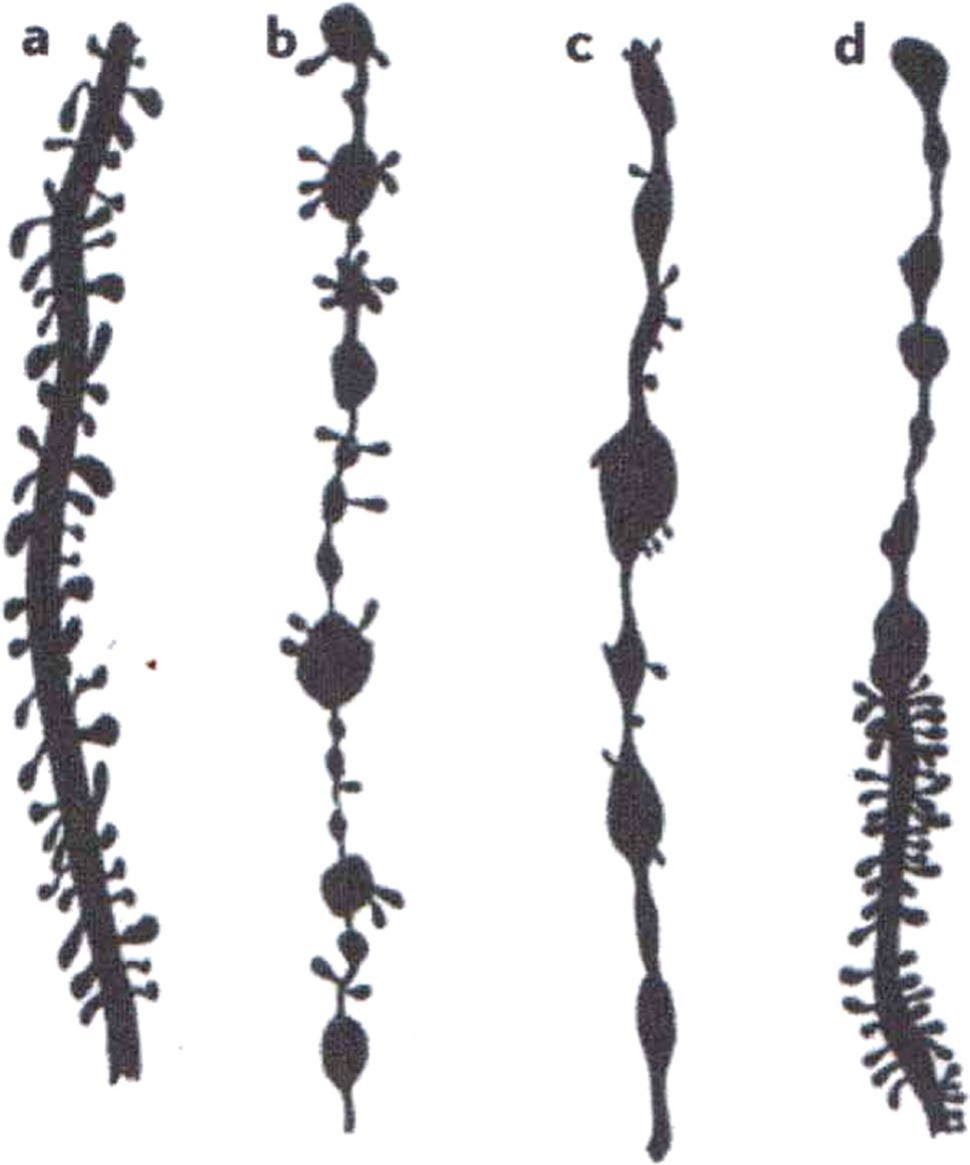
Changes in dendrites of pyramidal neurons in the guinea pig due to excessive electrical stimulation. a. spines of a normal dendrite, b-c: loss of spines and beaded appearence of dendrites in experimental animals. Golgi technique [54] Reproduced from De Felipe (2002)

Black [51] mentions the great support that the amoeboid theory received at the time. She cites Van Gieson in a 1898 review who “spoke of the neuron as a tiny octopus with the power of movement over its tentacles”. The evidence for the theory, however, could not be confirmed. In his [55] review, Bawden showed that the observed changes in the dendrites were artifacts of the Golgi method. Still, modern studies cited by DeFelipe (2002) and Gonzáles Burgoz [56] described the changes in density and shape of dendritic spines. “The studies on visual deprivation indicated that the formation and maintenance of spines depend on synaptic activity and that they can be modulated by sensory experience. By contrast, the studies on mental retardation identified changes in the morphology and density of spines that they might alter synaptic inputs to pyramidal neurons” (deFelipe, 2002). Amoeboid movements of axons, establishing new connections during learning in adults, recently were found by Boele et al. [57]. They observed an increased mumber of mossy fiber collateral terminals in the cerebellar nucleus involved in eyeblink conditioning in mice. In their experiments, they used a tone as the conditional stimulus that is transferred by auditory mossy fibers from the lateral pontine nuclei. The increase in number of the collateral terminals is positively correlated with the amplitude of the conditioned eyelid response.

It seems that the amoeboid theory was almost forgotton since the final decade of the nineteenth century. However, according to Jelgersma [33] it was generally accepted at the time. It is possible, therefore, that he did not refer to the original authors of the theory, because their contributions were general knowledge at the time. In general, in his publication he refers to the literature only sparsely. The customs of citing previous work may have been different in this period, but still, Jelgersma’s silence on the subject remains unexplained. Nonetheless, this paper was written to honour Jelgersma for proposing a theory of cerebellar function that certainly presages today’s concepts of cerebellar learning and plasticity.
